# Effect of duration of diabetes on bone mineral density: a population study on East Asian males

**DOI:** 10.1186/s12902-018-0290-y

**Published:** 2018-09-05

**Authors:** Miso Jang, Hyunkyung Kim, Shorry Lea, Sohee Oh, Jong Seung Kim, Bumjo Oh

**Affiliations:** 1 Department of Family Medicine and Center for Cancer Prevention and Detection, Hospital, National Cancer Center, 323, Ilsan-ro, Ilsandong, Goyang-si, Gyeonggi-do 10408 Republic of Korea; 2 Department of Family Medicine, DDH Hospital, 60, Hi park 2-ro, Ilsanseo-gu, Goyang-si, Gyeonggi-do 10234 Republic of Korea; 3 Center for Health Promotion, Cheil General Hospital, 17, Seoae-ro 1-gil, Jung-gu, Seoul, 04619 Republic of Korea; 4 Department of Biostatistics, SMG-SNU Boramae Medical Center, 20, Boramae-ro 5-gil, Dongjak-gu, Seoul, 07061 Republic of Korea; 5 Department of Family Medicine, SMG-SNU Boramae Medical Center, 20, Boramae-ro 5-gil, Dongjak-gu, Seoul, 07061 Republic of Korea

**Keywords:** Diabetes mellitus, Prediabetic state, Bone mineral density, Dual-energy X-ray absorptiometry, Osteoporosis, Korea National Health and nutritional examination survey

## Abstract

**Background:**

The aim of the present study is to evaluate the association between BMD and type 2 DM status in middle-aged and elderly men. To investigate a possible correlation, the present study used the BMD dataset of the Korea National Health and Nutrition Examination Survey (KNHANES) from 2008 to 2011.

**Methods:**

In total, 37,753 individuals participated in health examination surveys between 2008 and 2011. A total of 3383 males aged ≥50 years were eligible. They underwent BMD measurement through dual-energy X-ray absorptiometry (DXA). The fasting plasma glucose and insulin levels of participants were also measured.

**Results:**

Men with prediabetes and diabetes had significantly higher mean BMD at all measured sites than control men did, irrespective of DM status. This was confirmed by multivariable linear regression analyses. DM duration was an important factor affecting BMD. Patients with DM for > 5 years had lower mean BMD in the total hip and femoral neck than those with DM for ≤5 years. Per multivariable linear regression analyses, patients with DM for > 5 years had significantly lower mean BMD at the femoral neck than those with DM ≤5 years.

**Conclusions:**

DM duration was significantly associated with reduced femoral neck BMD.

## Background

Type 2 diabetes mellitus (DM) is a common metabolic disease with an increasing worldwide prevalence rate of 8.3% [1], and with 11% among Korean male adults. Considering that more than 9 million osteoporotic fractures are recorded annually worldwide, osteoporosis is a significant contributor to morbidity and lost life years globally [2]. Osteoporosis and type 2 DM share many common characteristics in that they are both chronic diseases with an increasingly global medical burden.

Although individuals with type 1 DM show decreased bone mass density (BMD), those with type 2 DM often have normal or even slightly elevated BMD compared with an age-matched control population [3]. Bone fragility results from decreased bone mineral mass and alterations in bone microstructure. Multiple mechanisms can contribute to increased fractures in type 2 DM patients. Glucose toxicity, lack of insulin and other factors affects bone metabolism. A substantial number of studies examined the association between type 2 DM and fracture risk [4, 5]. Longer type 2 DM duration increases diabetic complications, insulin usage, and fracture risk and results in inadequate glucose control. Clinically, assessing the bone microstructure of type 2 DM patients is difficult because CT or MRI should be used [3]. Therefore determining the BMD is the best approach for now.

The prevention of fractures is an important goal for studies concerning older adults. Many studies focus on osteoporosis in women. However, as many as one in four men aged > 50 years will develop at least one osteoporosis-related fracture in his lifetime, highlighting the need for more studies on osteoporosis in men [6]. One in three men die in within a year after a hip fracture, another one in three experience a subsequent fracture again [7]. Generally, men have worse smoking and alcohol drinking habits and higher risk of fall than women [8], which may contribute to bone health deterioration. Furthermore, men are approximately 70% less frequently screened for osteoporosis than women [9]. For individuals aged ≥50 years in Korea, the prevalence rates of osteoporosis and osteopenia are 7.3% and 38.0% in men and 46.5% and 48.7% in women, respectively [10]. In another Korean study, only 7.6% of men found out that they had osteoporosis, and only 5.7% of them had their disease treated [11].

In light of public health, male osteoporosis associated with type 2 DM should be carefully considered. The present study aimed to assess the association between BMD and type 2 DM status by considering several confounding factors such as age, body mass index (BMI), and fasting insulin and glucose level. We hypothesized that participants with longer duration of type 2 DM may have lower BMD because of poor disease control and insulin deficiency.

## Methods

### Study design and participants

**Fig. 1:**
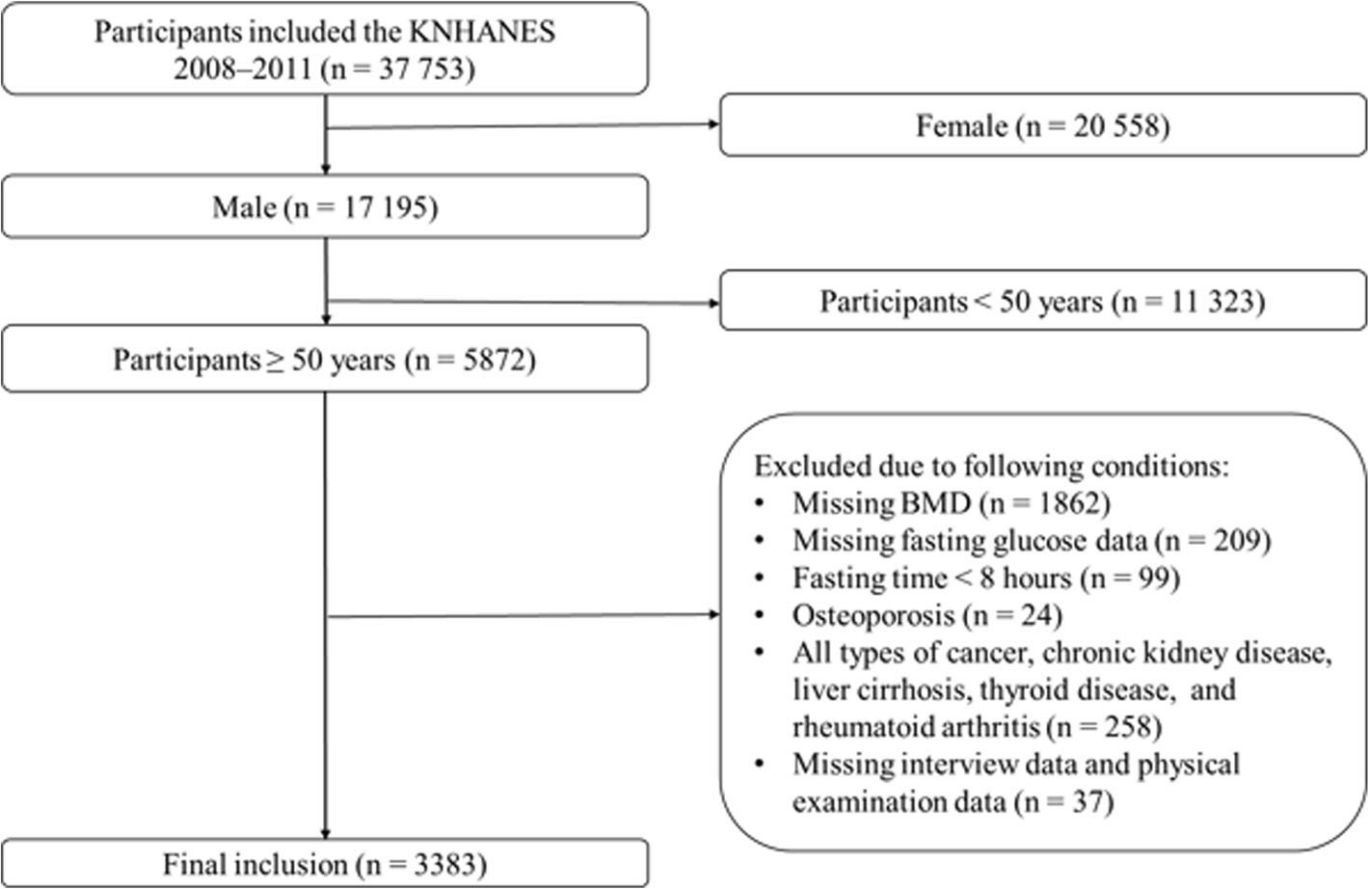
Flow diagram for identification the study population. A total of 3380 participants were finally included. *KNHANES*, Korea National Health and Nutritional Examination Survey; *BMD*, Bone Mass Density

The Korea National Health and Nutritional Examination Survey (KNHANES) is a nationwide survey representing the non-institutionalized civilian population of South Korea. The Division of Health and Nutritional Survey of the Korea Centers have periodically conducted it for Disease Control and Prevention (KCDCP) since 1998. The data from KNHANES is open data for research purposes. A complex, stratified, multilevel probability sampling design was used, and sampling units were selected based on geographical area, age and sex [12]. Each sampled participant is assigned a numerical sample weight that measures the number of populations represented by that specific participant [12]. A complex sampling design and sample weights facilitate the production of nationally representative data [13]. The KNHANES consists of three components: a health interview, a nutrition survey, and a health examination. Until the first half of 2008, data were gathered through household interviews and direct standardized physical examinations conducted at simple checkup centers at city government offices and town halls [14]. From the second half of 2008 to 2011, the survey was conducted by introducing mobile inspection vehicles. In July 2008, the whole-body dual-energy x-ray absorptiometry (DXA) survey was newly introduced and tested until May 2011. People who were tested before July 2008 and after May 2011 have not received the DXA. From 2008 to 2011, the KNHANES included 37753 individuals. Of the 5872 male participants who were aged ≥50 years, 2489 were eliminated from the study based on the following exclusion criteria: missing data, fasting time of < 8 h, taking prescription medication for osteoporosis, and the questionnaire answered with having been diagnosed and treated by physicians for the conditions affecting bone metabolism, such as all types of cancer, chronic kidney disease, liver cirrhosis, thyroid disease, or rheumatoid arthritis (Fig. 1). After all exclusions, 3383 participants were finally included in this analysis.

### Associated factors

As described previously [12], the demographic and behavior variables were age, monthly house income, smoking (never, past, or current smoker), alcohol drinking (grams of alcohol per day), and physical activity (low, moderate, or high). The average alcoholic beverage intake was assessed by self-reported questionnaire and then converted to the amount of pure alcohol (in grams) consumed per day. Physical activity was quantified as the metabolic equivalent of task (MET) minutes per week calculated using the Korean version score calculation of the short-form International Physical Activity Questionnaire. Therefore, physical activity levels were then classified as low (< 600 MET minutes per week), moderate (600 ≤ − < 3000 MET minutes per week), or high (≥3000 MET minutes per week) [12].

Standardized techniques and equipment were used to measure height and weight, and body mass index (BMI) was calculated by dividing body weight by the square of height (kg/m^2^) [14]. Blood pressure (BP) was measured by a standard method using a sphygmomanometer in a sitting position. The following definition for hypertension was obtained from the Division of Health and Nutritional Survey under the KCDCP: either by a self-reported history of hypertension diagnosis and current usage of antihypertensive drug or by ≥140 mmHg systolic BP or ≥ 90 mmHg diastolic BP.

### Laboratory examinations

During the survey, in the morning, blood samples were collected and analyzed by a certified central laboratory. Plasma total cholesterol (milligrams per deciliter), high-density lipoprotein (HDL) cholesterol (milligram per deciliter), triglycerides (milligrams per deciliter) and fasting glucose levels (milligrams per deciliter) were enzymatically measured using a Hitachi Modular 7600 automatic analyzer (Hitachi Ltd., Tokyo, Japan). The fasting insulin (micro-International units per milliliter) and serum vitamin D (nanograms per milliliter) concentrations were measured using immunoradiometric and radioimmunoassays using a gamma-counter (1470 Wizard; PerkinElmer, Waltham, MA, USA). The direct measurement of low-density lipoprotein (LDL) cholesterol was limited, and it was mainly calculated using the Friedewald equation (i.e., LDL cholesterol = total cholesterol – HDL cholesterol – triglyceride/5) [15]. Insulin resistance was evaluated using the homeostasis model assessment: estimated insulin resistance (HOMA-IR) index (i.e., HOMA-IR = [fasting plasma glucose] × [fasting plasma insulin] × 0.055 /22.5) and homeostasis model assessment: estimated beta cell function (HOMA-beta) index (i.e., HOMA-beta = 20 × [fasting plasma insulin / ([fasting plasma glucose] × 0.055–3.5)] [16].

### Assessment of BMD

The whole-body dual-energy x-ray absorptiometry (DXA) survey begin in July 2008 and ended in May 2011. The BMDs of the lumbar spine, left total hip, and left femoral neck, and body composition including percent fat mass, were measured using DXA (Discovery-W; Hologic Inc., Waltham, MA, USA). As described previously [14], for the lumbar spine, the mean value of BMDs of L1–L4 was selected for analysis according to the recommendation of the International Society for Clinical Densitometry [17]. The BMD of the right femur was used when the left femur could not be measured (e.g., postoperative state, fracture, deformity, or malformation). The technicians performing the BMD measurements tested the precision based on duplicate or triplicate measurements in 30 or 15 participants, respectively. The precision error was calculated as the percentage coefficient of variation (CV %). The precision errors allowed for the total hip, femur neck, and lumbar vertebrae were 1.8%, 2.5%, and 1.9%, respectively [14].

### Assessments and definition of DM/prediabetes and duration of DM

The following definitions were obtained from the Division of Health and Nutritional Survey of the KCDCP. DM was defined by either a self-reported history of DM diagnosis and current usage of insulin or oral hypoglycemic agents, or by a ≥ 126 mg/dL fasting blood glucose level or ≥ 6.5% glycated hemoglobin (HbA1c) level. Prediabetes was defined as ≥100–125 mg/dL fasting blood glucose, or ≥ 5.7–6.5 HbA1c levels. DM duration was calculated by subtracting the age at the time of DM diagnosis from the age at the time of the investigation. Data without time-diagnosed diabetes were excluded.

### Statistical analysis

Statistical analyses were carried out using STATA 14.0 (StataCorp, College Station, TX, USA) with the SVY commands to account for the complex sampling design and include sample weight, which enabled the results to represent the entire national male population > 50 years [13]. All continuous data were presented as the mean ± standard error (SE). All categorical data were presented as numbers and percentages. To compare the participants’ characteristics of the study among control, groups with prediabetes and DM, the analysis of variance (ANOVA) test and χ^2^ test for continuous and categorical variables, respectively, were used. Multivariable linear regression analyses were performed to determine whether the BMD at each measured site differed based on the DM status and duration. In model 1, we adjusted for major factors, such as age and BMI. Model 2 was also adjusted for alcohol consumption; serum vitamin D concentration; smoking status; triglyceride levels, HDL cholesterol levels and total cholesterol levels, hypertension diagnosis, physical activity level, and HOMA-IR index scores. LDL cholesterol was not included as a covariate in model 2 because a significant collinearity was found between LDL and total cholesterol levels. Bonferroni’s correction was performed for comparisons between the control group and groups with prediabetes and DM. The reported probability values are two-sided, and results with a *p*-value < 0.05 were considered statistically significant.

## Results

### Baseline characteristics of the participants based on type 2 DM status

Among all participants aged ≥50 years of the 2008–2011 KNHANES, 1037 (29.76%) men had prediabetes, and 644 (18.57%) men had DM. Table 1 shows the baseline characteristics of the control, participants with prediabetes and DM. Patients with diabetes were slightly older than men in other groups. Men who had prediabetes and diabetes were more likely to be overweight or obese have hypertension; have higher fasting glucose levels, HOMA-IR index scores, HDL and LDL cholesterol levels, triglyceride levels, and alcohol consumption; and have lower HOMA-beta index scores, than those in the control group (*p* < 0.05). The control, prediabetic, and diabetic groups differed in terms of BMD based on the World Health Organization’s criteria.

**Table 1:** Weighted baseline characteristics of study participants from the KNHANES (2008–2011)

	Men (*n* = 3383, *N* = 4 370 109)			*p*-value
	Normal	Prediabetes	Diabetes	
	*n*=1702(51.67)^b^	*n*=1037(29.76)^b^	*n*=644(18.57)^b^	
Age (years)	60.46 ± 0.27	60.63 ± 0.32	61.59 ± 0.41	0.03
Weight (kg)	65.40 ± 0.29	68.25 ± 0.40	68.97 ± 0.51	<0.001
Height (cm)	167.11 ± 0.18	167.46 ± 0.24	166.72 ± 0.29	0.53
BMI (kg/m2)	23.39 ± 0.09	24.29 ± 0.11	24.78 ± 0.16	<0.001
Fat mass (%) (*n* = 3298)	21.74 ± 0.15	23.03 ± 0.18	23.49 ± 0.25	<0.001
Low income (*n* = 3342) *n*, (%)	484 (11.87)	246 (5.83)	217 (5.53)	0.003
Physical activity *n*, (%)^a^				
low	429 (25.90)	259 (23.59)	159 (24.23)	
moderate	663 (36.97)	417 (40.40)	272 (43.13)	
high	608 (37.12)	360 (36.01)	211 (32.64)	
	1700	1036	642	0.65
Smoking history *n*, (%)				
never	305 (18.11)	191 (16.41)	101 (14.28)	
past	817 (45.87)	525 (48.27)	327 (47.22)	
current	580 (36.02)	321 (35.33)	216 (38.50)	
	1702	1037	644	0.397
alcohol consumption (g/day)	13.42 ± 0.56	16.24 ± 0.77	15.23 ± 1.14	0.043
hypertension^a^	848(44.13)	705(34.1)	446(21.7)	<0.001
fasting glucose (mg/dl)	90.99 ± 0.17	107.03 ± 0.27	141.38 ± 1.96	<0.001
fasting insulin (IU/mL) (*n* = 2927)	8.74 ± 0.13	10.40 ± 0.19	10.83 ± 0.33	<0.001
HbA1c (*n* = 1001)	5.45 ± 0.02	5.91 ± 0.03	7.21 ± 0.07	<0.001
HOMA-IR (*n* = 2927)^a^	1.95 ± 0.03	2.75 ± 0.05	3.74 ± 0.12	<0.001
HOMAbeta (*n* = 2927)^a^	120.59 ± 1.89	86.18 ± 1.53	58.97 ± 6.70	<0.001
DM duration (years) (*n* = 505)			8.43 ± 0.34	
total cholesterol (mg/dl)	186.99 ± 1.09	192.80 ± 1.40	181.16 ± 2.00	0.168
HDL-C (mg/dl)	45.26 ± 0.36	45.72 ± 0.43	42.27 ± 0.49	<0.001
LDL-C (mg/dl)	111.77 ± 1.15	111.11 ± 1.46	99.98 ± 2.03	<0.001
Triglyceride (mg/dl)	149.80 ± 4.87	179.85 ± 5.37	194.53 ± 7.85	<0.001
serum vitamin D (ng/mL)	21.38 ± 0.29	21.17 ± 0.34	20.52 ± 0.41	0.062

^a^The data are presented as n (%) or the means ± standard error (SE). *n* = unweighted; *N* = the size of Korean population estimated using sample weights; physical activity level = low (<600 MET minutes per week), moderate (≥600-<3000 MET minutes per week), high (≥3000 MET minutes per week); hypertension = diagnosis and current usage of antihypertensive drug or ≥140 mmHg systolic BP, or ≥90 mmHg diastolic BP; HOMA-IR = [fasting plasma glucose] × [fasting insulin] ×0.055/22.5; HOMA-beta = 20 × [fasting plasma insulin / ([fasting plasma glucose] × 0.055-3.5); HbA1c = glycated hemoglobin; LDL-C = low-density lipoprotein cholesterol; HDL-C = high-density lipoprotein cholesterol

^b^The prevalence (%) are presented weighted value

### Association between BMD and DM status

The BMDs at various measured sites in the control, prediabetic, and diabetic groups were compared. Men with prediabetes and diabetes had higher lumbar spine and total hip BMDs than controls in the crude analysis. The prediabetic group had higher femoral neck BMDs than the control group. No significant differences in three BMD sites were found between the prediabetic and diabetic groups. In model 1, after adjusting for age and BMI, the prediabetic group had higher lumbar spine and total hip BMDs than the control group. After further adjustments for all clinically relevant covariates (model 2), the men with DM and men with prediabetes had higher lumbar spine, total hip, and femoral neck BMDs than controls. Similarly, no significant difference at three BMD sites were found between the prediabetic and diabetic groups in both models 1 and 2 (Table 2).

**Table 2:** Comparison of BMDs (g/cm²) at various measured sites

	Men (*n* = 3383, *N* = 4 370 109)^c^			Normal vs. preDM	Normal vs. DM	PreDM vs. DM
	Normal	PreDM	DM			
	*n* = 1702 (51.67%)	*n* = 1037 (29.76%)	*n* = 644 (18.57%)	*p*-value	*p*-value	*p*-value
Crude						
lumbar spine (*n* = 3215)	0.928 ± 0.004	0.966 ± 0.006	0.973 ± 0.008	<0.001*	<0.001*	0.478
total hip	0.924 ± 0.003	0.951 ± 0.005	0.950 ± 0.006	<0.001*	<0.001*	0.872
femoral neck	0.751 ± 0.003	0.768 ± 0.005	0.762 ± 0.006	0.004*	0.091	0.448
Model 1^a^						
lumbar spine	0.923 ± 0.001	0.962 ± 0.001	0.967 ± 0.002	0.001*	0.006*	0.994
total hip	0.911 ± 0.002	0.940 ± 0.002	0.936 ± 0.003	0.014*	0.226	0.434
femoral neck	0.738 ± 0.001	0.757 ± 0.002	0.749 ± 0.002	0.228	0.702	0.222
Model 2^b^						
lumbar spine	0.922 ± 0.001	0.967 ± 0.002	0.969 ± 0.002	<0.001*	0.001*	0.765
total hip	0.912 ± 0.002	0.944 ± 0.002	0.939 ± 0.002	<0.001*	<0.001*	0.316
femoral neck	0.738 ± 0.002	0.760 ± 0.002	0.752 ± 0.003	0.046	0.034	0.707

^a^Model 1: adjusted for age and BMI

^b^Model 2: adjusted for age, BMI, alcohol consumption, serum vitamin D, smoke status, triglyceride, HDL-cholesterol, total cholesterol, hypertension, physical activity, and HOMA-IR

^c^The data are presented as means ± SE. *n* = unweighted; *N* = the size of Korean population estimated using sample weights

*P<0.0167 compared to 3 groups by Bonferroni’s correction

### Baseline characteristics of the participants based on DM duration

Table 3 shows the baseline characteristics of the participants with diabetes duration of ≤5 years and those with a disease duration of > 5 years. A total of 505 people were identified for type 2 DM duration: 235 participants with a disease duration of ≤5 years and 270 with longer DM duration. No significant differences between the two groups were found in terms of insulin-associated factors, BMI, fat mass, total cholesterol levels, and HDL and LDL cholesterol levels. However, longer DM duration participants were slightly older with a lower percentage of current smokers and had higher fasting glucose and HbA1c levels than those with shorter DM duration.

**Table 3:** Weighted baseline characteristics of the diabetes group based on diabetic duration

	Men (*n* = 505, *N* = 618 430)		*p*-value
	DM duration ≤ 5 years	DM duration > 5 years	
	*n* = 235 (51.01)^b^	*n* = 270 (48.99)^b^	
Age (years)	60.45 ± 0.60	62.79 ± 0.64	0.008
Weight (kg)	70.25 ± 0.90	68.06 ± 0.69	0.054
Height (cm)	167.24 ± 0.56	166.33 ± 0.43	0.199
BMI (kg/m2)	25.06 ± 0.26	24.56 ± 0.21	0.132
Fat mass (%) (*n* = 495)	23.66 ± 0.43	23.58 ± 0.37	0.879
Low income (*n* = 497) n, (%)	79 (26.95%)	93 (34.15%)	0.124
Physical activity n, (%)^a^			
low	59 (21.82)	66 (27.29)	
moderate	95 (44.34)	115 (40.35)	
high	80 (33.84)	88 (32.38)	
	234	269	0.5099
Smoker (%)			
never	45 (18.62)	40 (12.41)	
past	100 (34.80)	153 (55.73)	
current	90 (46.58)	77 (31.86)	
	235	270	0.0005
alcohol (g/day)	15.96 ± 1.78	12.90 ± 1.61	0.204
Hypertension (%)^a^	34.98	30.29	0.201
fasting glucose	134.42 ± 2.98	144.06 ± 3.82	0.047
fasting insulin (*n* = 432)	10.24 ± 0.49	10.66 ± 0.45	0.528
HbA1c (*n* = 488)	6.97 ± 0.09	7.54 ± 0.11	<0.001
HOMA-IR (*n* = 432)^a^	3.39 ± 0.21	3.70 ± 0.16	0.243
HOMAbeta (*n* = 432)^a^	67.94 ± 5.39	51.60 ± 17.91	0.383
total cholesterol (mg/dl)	175.71 ± 2.79	179.53 ± 3.21	0.37
HDL-C (mg/dl)	42.53 ± 0.78	41.79 ± 0.76	0.498
LDL-C (mg/dl)	98.73 ± 2.56	100.91 ± 2.58	0.549
Triglyceride (mg/dl)	172.25 ± 9.44	182.15 ± 9.12	0.365
serum vitamin D (ng/mL)	20.27 ± 0.55	20.60 ± 0.59	0.69

^a^The data are presented as n (%) or the means ± standard error (SE). *n* = unweighted; *N* = the size of Korean population estimated using sample weights; physical activity level = low (<600 MET minutes per week), moderate (≥600-<3000 MET minutes per week), high (≥3000 MET minutes per week); hypertension = diagnosis and current usage of antihypertensive drug or ≥140 mmHg systolic BP, or ≥90 mmHg diastolic BP; HOMA-IR = [fasting plasma glucose] × [fasting insulin] ×0.055 /22.5; HOMA-beta = 20 × [fasting plasma insulin / ([fasting plasma glucose] × 0.055-3.5); HbA1c = glycated hemoglobin; LDL-C = low-density lipoprotein cholesterol; HDL-C = high-density lipoprotein cholesterol

^b^The prevalence (%) are presented weighted value

### Association between BMD and DM duration

The BMDs at various measured sites were compared between the participants with DM duration of ≤5 years and those with disease duration of > 5 years. The group with longer DM durations had lower total hip and femoral neck BMDs than those with shorter DM durations in the crude analysis. The strength of the association between total hip BMD and DM duration declined after further adjustments; however, the group with longer DM durations had lower femoral neck BMDs than those with shorter DM durations in both models 1 and 2 (Table 4).

**Table 4:** Comparison of BMDs (g/cm²) at various measured sites based on diabetes duration

	Men (*n* = 505, *N* = 618 430)^c^		*p*-value
	diabetic duration ≤ 5 years	diabetic duration > 5 years	
	*n*=235(51.01)	*n*=270(48.99)	
Crude			
lumbar spine (*n* = 481)	0.979 ± 0.013	0.977 ± 0.012	0.906
total hip	0.968 ± 0.009	0.940 ± 0.008	0.026*
femoral neck	0.786 ± 0.009	0.746 ± 0.008	0.001*
Model 1^a^			
lumbar spine	0.976 ± 0.002	0.976 ± 0.002	0.914
total hip	0.953 ± 0.004	0.930 ± 0.003	0.258
femoral neck	0.770 ± 0.004	0.735 ± 0.003	0.026*
Model 2^b^			
lumbar spine	0.976 ± 0.003	0.977 ± 0.003	0.896
total hip	0.955 ± 0.005	0.933 ± 0.004	0.337
femoral neck	0.773 ± 0.004	0.738 ± 0.004	0.018*

^a^Model 1: adjusted for age and BMI

^b^Model 2: adjusted for age, BMI, alcohol consumption, serum vitamin D, smoke status, triglyceride, HDL-cholesterol, total cholesterol, hypertension, physical activity, and HOMA-IR

^c^Data are presented as means ± SE. *n* = unweighted; *N* = the size of Korean population estimated using sample weights.

*Statistically significant (*p*<0.05) on the basis of the multivariable linear regression test

## Discussion

In the present study, the prediabetic and diabetic groups had higher mean BMDs at all measured sites than the control group. The BMDs in men with prediabetes were similar to those in men with diabetes in all cases. However, men with diabetes with a disease duration of > 5 years had lower mean femoral neck BMDs than those with a disease duration of ≤5 years after adjustment for all clinically relevant covariates.

Asian people develop diabetes at a lower degree of obesity and at younger ages and experience chronic diabetic complications [18]. In addition, some studies in Japan [19] and Korea [20] have showed that Asian patients with type 2 DM have lower BMI and decreased β-cell function compared with European and American patients. Current study showed that each groups had similar BMI in overweight range, but not obesity. Therefore a study on the relationship between type 2 diabetes and osteoporosis in Asian population would be good in isolating the effect of obesity.

The association between BMD and type 2 DM remains unclear. However, the results of the current study are consistent with those of previous studies in that DM patients have higher BMDs [21–23]. This study showed that men with prediabetes had higher BMDs than controls. Moreover, in this study, men with prediabetes and DM showed similar fasting insulin levels. Excessively high insulin level in the blood has been reported to be associated with increased bone mass [24] because of the anabolic effects of insulin [25] and increase in free sex hormone levels [26]. Although prediabetes is not considered a disease, insulin resistance in prediabetes will affect bone mass and microstructure [12].

Bone fragility results from decreased BMD and alterations in bone microstructure [3]. Assessing the macrogeometry of cortical bone and the microarchitecture of the trabecular bone is difficult owing to the use of quantitative CT or MRI. In clinical setting, the gold standard of bone strength measurement is DXA and BMD remains a significant predictor of fracture risk in type 2 DM, that is, independent of trabecular bone score and DM itself [27].

Elevated fasting insulin levels play a key role in DM development, and they mostly result in increased bone mass. In complicated conditions, such as advanced type 2 DM, elevated insulin levels have an unexpected effect. As insulin resistance increases, the fasting insulin levels are inversely related to BMD, and this relationship becomes more significant as the degree of insulin resistance increases [12]. In advanced type 2 DM requiring insulin, pancreatic ß- cell function certainly decreased. However, the exact timing of this phenomenon remains to be determined. A Korean prospective cohort study [20] reported on the role of ß-cell dysfunction on DM development, focusing on Asian populations. Insulin levels possibly increased and then decreased at some point, and the bone density may have become weak at this point.

In the present study, insulin levels were similar in prediabetes and diabetes and in two groups with different duration of disease. It was significantly different that fasting glucose levels in prediabetes and diabetes, and HbA1c levels between men with diabetes duration of ≤5 years and those with diabetes duration > 5 years. High blood glucose induces formation of advanced glycation end-products (AGE), with negative effects on structural proteins such as type I collagen, the main bone matrix protein. AGE may also reduce bone strength by impairing bone formation [28]. Most of the recent studies have confirmed decreased levels of bone turnover markers in patients with DM [3]. The previous research on mechanisms of type 2 DM showed that action on bone with long-term high glucose levels could lower the turnover, resulting in unfavorable bone balance.

Researchers have generally neglected osteoporosis in men for some time, and many studies have focused on women as participants. One study showed that non-obese women with type2 DM had lower BMD than control participants matched for BMI [29]. In the present study, we investigated the correlation between BMD and BMI in the group with DM. Our results confirmed the absence of such a correlation. Obese patients with type 2 DM have increased BMD, and evidence indicates that older white women, but not men or black women, with diabetes exhibited more rapid bone loss at the femoral neck and total hip than those with normal glucose homoeostasis [30]. Type 2 DM has been associated with higher bone loss at the femoral neck than at the total hip in white women even after adjusting for weight loss. Although white women with type 2 DM had higher baseline BMDs, they still exhibited increased bone loss rate, particularly at the femoral neck, than those with normal glucose homoeostasis. This seemingly contradictory finding of higher cross-sectional BMD being associated with more rapid bone loss may reflect the net result of the positive effects of excessive weight and hyperinsulinemia on bones combined with the negative effects of longer diabetes duration [31].

Possible explanations for significant reduction of femoral BMD are existing literature on bone loss and cortical porosity. Pentosidine is the best-studied AGEs to date. The content of pentosidine in cortical and trabecular bone was higher in patients with femoral neck fractures than in age-matched controls [3]. These women with prior fractures have significantly lower femoral neck volumetric BMD, a trend towards larger bone volume and thinner cortices on quantitative CT, and higher serum levels of sclerostin than women with diabetes without fractures and nondiabetic controls with fractures(increases of 31.4% and 25.2%, respectively) [32].

Patients with type 2 DM have a significantly higher fracture risk than the general population [3, 5]. Men with type 2 DM have lower muscle mass and strength, contributing to the higher incidence of falls and fractures observed in type 2 DM patients [33]. Hip fracture is the most serious osteoporotic fracture, and our study shows that the femoral neck BMD was lower in the group with a DM duration of > 5 years than in those with a DM duration of ≤5 years. Previous studies showed that patients with type 2 DM have an increased fracture risk in the hip [5, 22]. Furthermore, another study revealed that women with a DM duration of ≥10 years have particularly high major osteoporotic and hip fracture risks [33].

This study used relatively large sample sizes representing national population-based data. The sample design and size were also estimated using the methods described in the KNHANES. Therefore, the results can be generalized to whole Korean diabetics. This study is the first to indicate decreased femoral neck BMD in long-time DM in Asia, which is consistent with the findings of previous studies. Current study demonstrated decreased BMD by DXA with duration of type 2 DM different from the earlier studies. However, several limitations should be considered when interpreting the results of this study. The intrinsic nature of cross-sectional design studies precludes this study from conclusively determining any potential causal relationships. The KNHANES was conducted annually, and the subjects were those who were not able to undergo DXA between 2008 and 2011, despite the introduction of DXA in 2008. This study did not consider the anti-diabetic medication in the group with DM; therefore, the effects of drugs are unknown. The reduction in BMD associated with type 2 DM duration requires further study. Five-year type 2 DM duration is a very short period to evaluate its effects on BMD. Moreover, femoral neck BMD decreased in participants with relatively short-term type 2 DM; thus, caution is needed when interpreting the results.

## Conclusions

This study aimed to assess the association between BMD and type 2 DM status in middle-aged or older men based on a nationwide survey. DM duration was significantly associated with reduced femoral neck BMD in men after adjusting for associated factors, such as age, BMI, and serum vitamin D level.

## Abbreviations

AGE: Advanced glycation end-products
BMD: bone mineral density
BMI: body mass index
BP: Blood pressure
CV: Coefficient of variation
DM: diabetes mellitus
DXA: Dual-energy X-ray absorptiometry
HbA1c: Glycated hemoglobin
HDL: High-density lipoprotein
HOMA-beta: Homeostasis model assessment: estimated beta cell function
HOMA-IR: Homeostasis model assessment: estimated insulin resistance
KCDCP: Korea Centers for Disease Control and Prevention
KNHANES: Korea National Health and Nutritional Examination Survey
LDL: Low-density lipoprotein
MET: Metabolic equivalent of task
